# Mapping and Optically Writing Nanogap Inhomogeneities in 1-D Extended Plasmonic Nanowire-on-Mirror Cavities

**DOI:** 10.1021/acsphotonics.4c01443

**Published:** 2024-12-10

**Authors:** Chetna Taneja, Eoin Elliott, G. V. Pavan Kumar, Jeremy J. Baumberg, Rohit Chikkaraddy

**Affiliations:** † NanoPhotonics Centre, Cavendish Laboratory, Department of Physics, University of Cambridge, JJ Thompson Avenue, Cambridge CB3 0HE, U.K.; ‡ Department of Physics, Indian Institute of Science Education and Research, Pune 411008, India; § School of Physics and Astronomy, University of Birmingham, Birmingham B15 2TT, U.K.

**Keywords:** gap plasmon modes, plasmonic nanocavity, picocavity, dark-field scattering, SERS

## Abstract

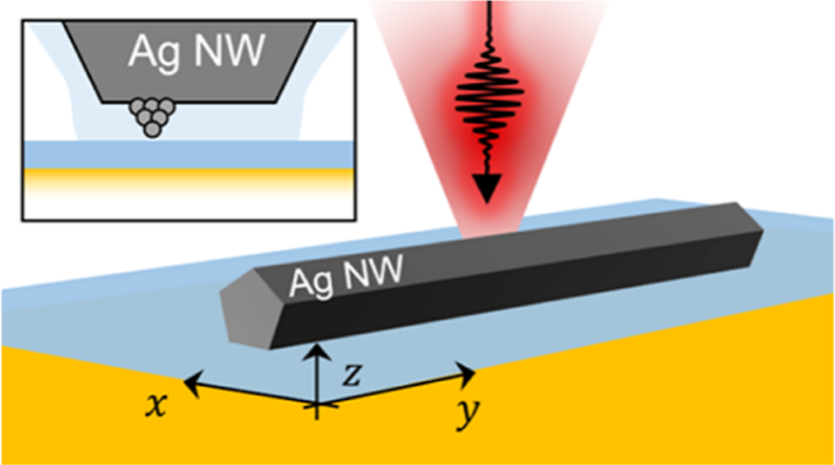

Tightly confined plasmons in metal nanogaps are highly sensitive to surface inhomogeneities and defects due to the nanoscale optical confinement, but tracking and monitoring their location is hard. Here, we probe a 1-D extended nanocavity using a plasmonic silver nanowire (AgNW) on mirror geometry. Morphological changes inside the nanocavity are induced locally using optical excitation and probed locally through simultaneous measurements of surface enhanced Raman scattering (SERS) and dark-field spectroscopy. The increasing molecular SERS intensity and corresponding redshift of cavity plasmon modes by up to 60 nm indicate atomic-scale changes inside the nanocavity. We correlate this to diffusion of silver atoms into the nanogap, which reduces the nanogap size and enhances the optical near-field, enhancing the SERS. These induced changes can be locally excited at specific locations along the length of the nanowire and remain stable and nonreversible. Polymer surface coating on the AgNW affects the power threshold for inducing atom migration and shows that strong polyvinylpyrrolidone (PVP)– Ag binding gives rise to higher power thresholds. Such extended nanogap cavities are an ideal system to provide robust SERS while withstanding high laser powers. These results provide insights into the inhomogeneities of NW nanocavities and pave the way toward spatially controlled NW lithography in ambient conditions.

## Introduction

The ability to optically trigger morphological changes on a metal surface at atomic scales has widespread impact in electrochemistry,^1,2^ photocatalysis,^3−5^ biosensing,^6,7^ and spintronics.^8,9^ Conventionally, transmission electron microscopy^10,11^ or scanning tunning microscopy (SEM)^12,13^ is used to track and image metastable metal surface atoms.^14−17^ These techniques, however, are quite slow, complex, and limited in their use at room temperature under ambient conditions. Tracking local atomic-scale nonreversible morphological changes in metallic nanostructures using noninvasive tools at room temperature is still lacking.

Recently, metal nanocavities have been utilized to induce atomic-scale surface protrusions on faceted gold (Au) nanoparticles (NP) placed on a mirror (NPoM).^18,19^ The nanoscale gap (0.1–10 nm) formed between the NP and the mirror strongly confines visible wavelength optical fields. Under illumination, the confined fields can deliver sufficient force for a single Au adatom to hop into the gap, creating “picocavities”. These changes result in enhanced near-fields and new transient vibrational lines from molecules sandwiched inside the gap.^18^ Separately from such picocavities, atomic restructuring due to surface atoms transiently lifting into the gap (termed “flares”) have also been studied.^20^ These picocavities are induced and detected optically under laser excitation at room temperature. Surprisingly, the power thresholds required to induce such changes are low (∼100 μW/μm^2^ or less, depending on the molecules in the gap).^21^ However, this makes it challenging to excite molecules within the nanocavity without observing unstable surface enhanced Raman scattering (SERS) signals. Recent studies have explored how the power thresholds for inducing atomic motion depend on molecules,^21^ temperature,^22^ and different facets of the metal nanostructures.^23^ Here, we show that nanowire-on-mirror nanocavities (NWoMs) support high power thresholds for inducing atomic migration inside the nanocavity. Additionally, the extended hotspot along the nanowire (NW) in this NWoM geometry allows for spatially controlled field confinement, not achievable with NP structures.

The quasi-one-dimensional silver NWoM is an intriguing geometry because it supports an extended nanogap along the >10 μm length of the silver nanowire (AgNW).^24,25^ Chemically prepared AgNWs are suited for these applications due to their ease of fabrication and single crystalline nature.^26,27^ The AgNW side surfaces^16^ are coated with 4–5 nm polyvinylpyrrolidone (PVP) which is used as a capping agent during chemical synthesis.^26,27^ PVP binds strongly to Ag via the oxygen atom in the pyrrole ring, thus forming a C–O–Ag complex.^28^ This results in smooth NW surfaces which then create nanocavities (of >5 nm nanogap spacing) with extremely precise spectral characteristics. Their long length allows for probing under different types of illumination and for observing the near-field along each individual 1-D plasmonic nanogap.^29^ In the past, characteristic directional surface plasmon polariton (SPP) waveguiding and localized electromagnetic hot-spots along the AgNW length have been shown to yield a wide range of applications including optical antennae,^30,31^ remote SERS,^100,32^ and spin–orbit coupling^33,34^ at the nanoscale.

Here, we utilize the NWoM to provide strong field confinement (or plasmonic hot-spots) inside the extended gap along the length of the NW, which induces atomic migration on the AgNW surface.^21^ These morphological changes modify the optical near-field inside the extended nanogap, detected through millisecond (ms) time evolution of SERS intensity arising from molecules sandwiched inside the nanocavity. The long cavity length allows us to probe the same cavity at different powers to estimate the threshold power for observing atomic migration in the gap.

## Results and Discussion

**Figure 1:**
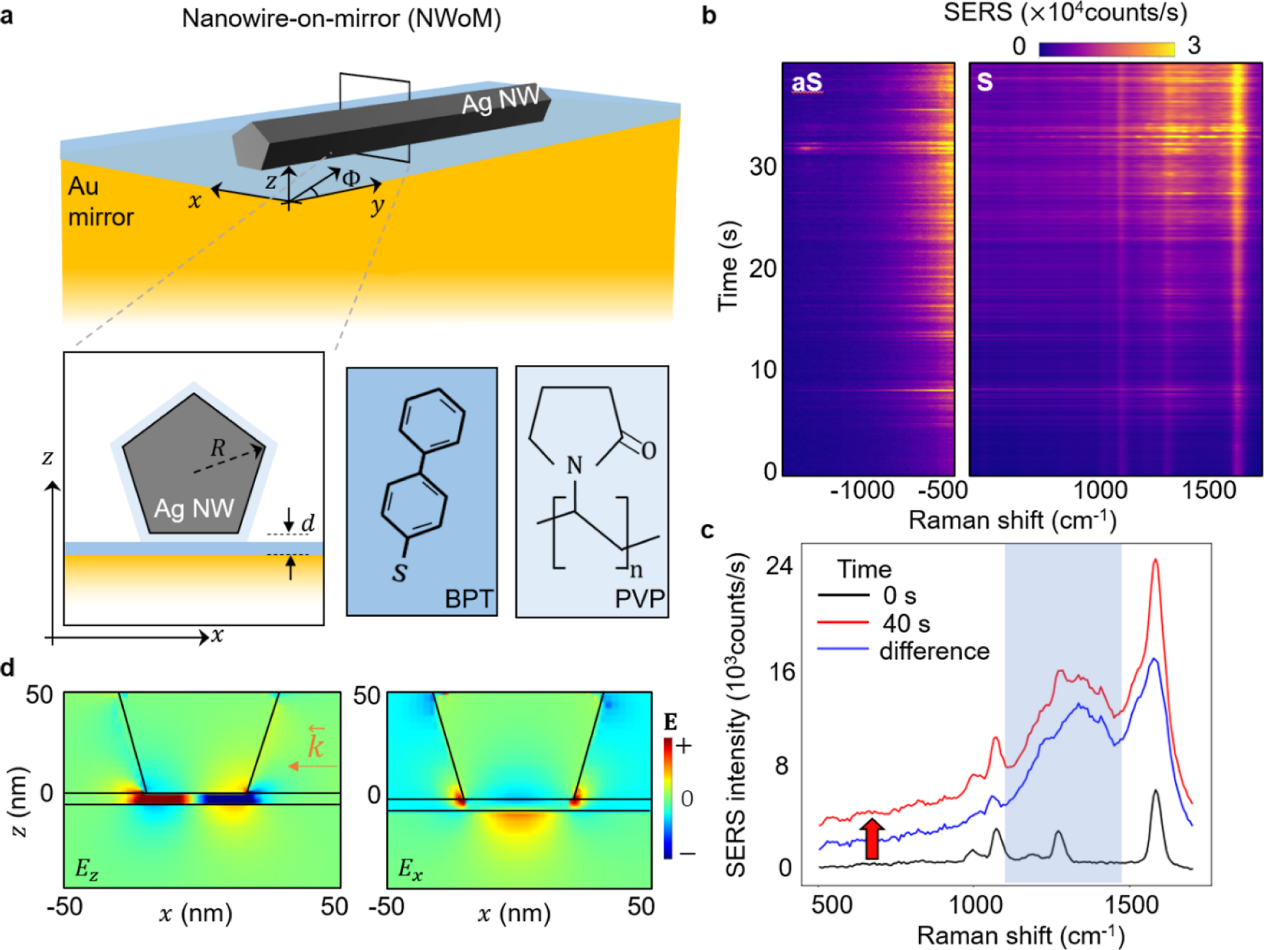
(a) Schematic of a chemically prepared silver nanowire (AgNW) extending along the *y* direction on a template-stripped gold (Au) mirror. Inset: cross-section of AgNW with polyvinylpyrrolidone (PVP) coating placed on a biphenyl-4-thiol (BPT) monolayer assembled on the Au mirror. PVP along with BPT scaffolds the nanogap *d* between the AgNW and Au mirror. (b) SERS time trace (Stokes on right, anti-Stokes on left) from the nanocavity when NWoM is excited with a 633 nm laser polarized perpendicular to the AgNW (along *x*-axis). The SERS intensity rises with time. Anti-Stokes intensities are multiplied by a factor of ×20 for comparison with the Stokes intensities. (c) SERS spectrum at *t* = 0 s (black curve), *t* = 40 s (red curve), and induced SERS (blue curve) calculated as the difference between red and black curves. Blue shading highlights new PVP - SERS peaks induced over 40 s. (d) Simulated near-field profiles of optical fields *E*_*z*_ and *E*_*x*_ in the NWoM nanocavity (*R* = 30 nm, *d* = 5 nm) excited using a Gaussian focus of λ = 633 nm polarized perpendicular to the AgNW (*z*-direction) with wave-vector direction along the *x*-axis (+ve *x*-axis to −ve *x*-axis).

The AgNWs are placed on a template-stripped Au film (Figure 1a) previously covered with a self-assembled monolayer (SAM) of biphenyl-4-thiol (BPT) molecules (Methods). This SAM of BPT molecules reproducibly forms on flat Au. Single crystalline AgNWs with pentagonal cross-section are chemically synthesized using a polyol process,^27^ giving a characteristic maximum radius *R* and are coated with PVP. The BPT monolayer and PVP coating together set the nanogap width *d* between the AgNW and Au film. The cross-sectional schematic of such a NWoM nanocavity with gap contents (BPT and PVP) is shown in Figure 1a.

The NWoM geometry is illuminated with a diffraction-limited focused laser of λ = 633 nm at a specific location (around the center of the NW) along its extended nanogap. Optical near-field profiles for a Gaussian focal spot (λ = 633 nm) evaluated with 2-D finite difference time domain (FDTD) simulations show strong field confinement inside the nanogap along the *z*-direction (Figure 1d). To match the experiments and simplify the simulations, the direction of the wavevector  is kept along the backward direction on the *x*-axis (from the positive *x*-axis to the negative *x*-axis). These confined fields result in intense SERS signals from the monolayer of BPT molecules placed inside the NWoM nanocavity (Figure 1b,c). The polarization of the excitation is kept along the perpendicular direction to the AgNWs (*x*-direction) to maximize the fields inside the nanogap. High SERS intensities enable us to watch dynamics on ms time scales with good signal-to-noise ratio during continuous laser exposure.

For laser excitation at optical powers *P* = 2.5 mW, SERS time traces at the excitation spot are recorded by taking 400 SERS spectra over times *t* = 40 s with an acquisition time of 100 ms for each. Time traces for Stokes (S) and anti-Stokes (aS) SERS intensities from BPT molecules inside the nanogap (Figure 1b) show three prominent characteristic BPT SERS lines^35^ at 1080 cm^–1^ (C–H rocking mode), 1256 and 1586 cm^–1^ (two in-plane stretches of benzene rings). Another feature to note is the broad background (flat baseline in Stokes side) resulting from inelastic light scattering from the electrons due to the light confinement inside the metal.^36−40^ The time trace reveals also the appearance of transient spectrally broad signatures called “flares”.^20,41^ Previously observed in NPoM nanocavities, these flares are optically induced by sub-Å lifting of the surface Au atomic layer from the facets of metallic nanoparticles.

After a prolonged illumination of 40 s, we observe an increase in all three BPT SERS signals as well as in the SERS background (red arrow) by >200%. The blue curve shows the difference between initial and final spectra and shows newly induced SERS peaks in addition to enhanced BPT SERS.

The extra SERS peaks in the 1150–1450 cm^–1^ region (overlapping with BPT SERS peak at 1256 cm^–1^) can be assigned to the interaction of PVP molecules (pyrrole ring) with Ag atoms.^28,42^ This Ag-PVP coordination modifies the nanogap cavity, giving rise to the BPT SERS enhancement. The observed growth in SERS intensities after prolonged illumination suggests the deforming nanocavity results in enhanced optical near-field intensities. For such metal–insulator–metal (MIM) nanocavities, the maximum field enhancement *E*_max_ inside a nanogap of thickness *d* and effective refractive index *n*_g_ is given by, *E*_max_^2^/*E*_0_^2^ ∝ *n*_g_*R*^2^/*d*^2^ where *E*_0_ is the incident optical field and *R* is the radius of the metallic nanostructure placed on the mirror forming the MIM nanocavity.^43,44^

Due to the complexity of directly accessing *d* and *n*_g_ in elongated NWoM nanocavities, we employ dark-field (DF) scattering techniques to map the plasmonic modes which confine the optical field inside the gap for NWoMs. These are very sensitive to local changes inside the nanogap.^29^ The resonance wavelengths of the modes reveal nanocavity morphology changes during laser exposure and the observed increases in molecular Raman emission.

**Figure 2:**
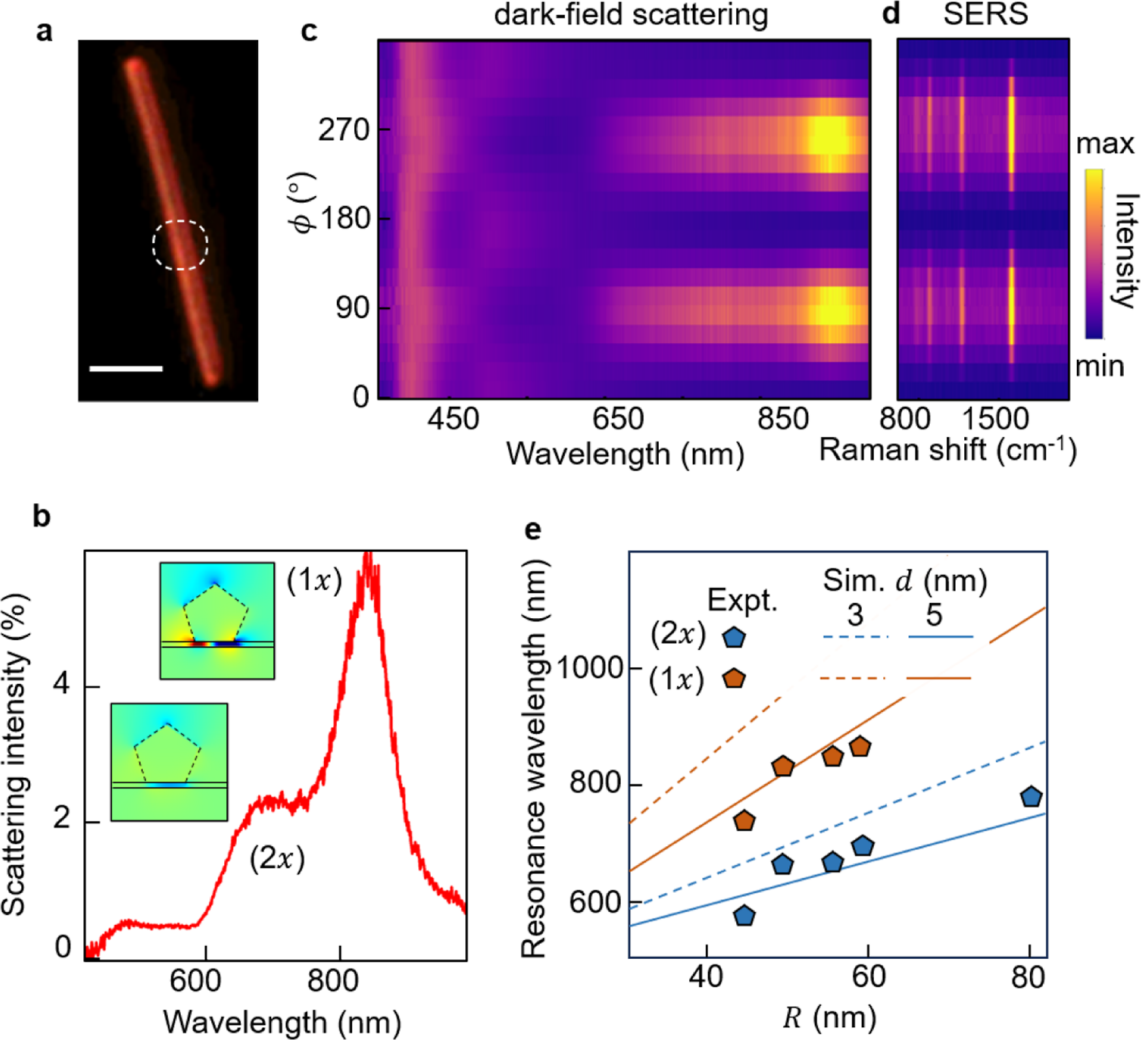
(a) Dark-field (DF) scattering image of NWoM geometry, scale bar: 1 μm. This geometry is excited with a white-light source for DF spectra and a laser (λ = 633 nm) for SERS, at the excitation position shown in white dashed circle. (b) DF scattering spectra of NWoM when polarization-analyzed along *x*-direction (ϕ = 90°), showing two plasmon modes termed (1*x*) and (2*x*). Inset shows simulated optical near-field along the perpendicular direction for (1*x*) and (2*x*) plasmon modes. (c) DF scattering spectra, and (d) SERS spectra, as a function of analyzer angle. (e) Simulated (1*x*, orange) and (2*x*, blue) resonance wavelengths vs NW radius *R*(dashed and solid lines for *d* = 3, 5 nm respectively). Orange and blue points show experimental resonance positions of (1*x*) and (2*x*) modes as a function of *R*.

The DF scattered light from individual NWoMs is resolved along the in-plane polarization angle (ϕ), defined as the angle between the analyzer axis and the AgNW long axis (*y*-axis) (Supporting Information, S1). The spectra reveal localized spectrally narrow resonances when analyzed perpendicular to the NW direction (*x*-axis, ϕ = 90°) (Figure 2b). Two characteristic modes are observed, labeled as (1*x*) and (2*x*). FDTD simulations are performed using a 2-D NWoM geometry (Methods). The near-field profiles (*E*_*z*_) for a AgNW with *R* = 30 nm for these two modes show strongly confined fields inside the nanogap between the AgNW and Au mirror, resulting from the gap plasmon modes of the NWoM geometry (inset of Figure 2b). For the (1*x*) mode at longer wavelengths, the optical field exhibits a node at the center of the facet, whereas the (2*x*) mode at shorter resonance wavelength has an intensity antinode in this position but two nodes near the facet edges. The (1*x*) and (2*x*) nomenclature thus denotes the number of nodes along the *x*-direction of the AgNW facet. Full polarization-resolved DF scattering spectra vs ϕ (Figure 2c) shows that gap plasmon modes are dominantly polarized perpendicular to the long-axis of the AgNW. By contrast, when analyzed along the length of the AgNW (*y*-axis, ϕ = 0°), the DF spectrum shows a broad resonance. These (1*x*) and (2*x*) modes elicit SERS from BPT molecules sandwiched inside the cavity. Full polarization-resolved DF scattering spectra are compared to the equivalent SERS spectra (Figure 2d). The intensity for SERS peaks from BPT molecules is maximized along the perpendicular direction to the AgNW long axis, which implies that SERS out-couples into the far-field by coupling to the gap plasmon mode with the same polarization signatures. Hence, for all further DF studies in these nanocavities, we measure DF scattering spectra analyzed perpendicular to the NW direction.

The confined near-field of these modes tunes the resonance position with *R* and gap thickness *d*. The resonance tuning of (1*x*) and (2*x*) modes as a function of *R* for *d* = 3, 5 nm is given in Figure 2e, compared to simulations. As predicted from increasing gap capacitance, (1*x*) and (2*x*) modes redshift as gap thickness decreases,^45^ consistent with simulations. Since both (1*x*) and (2*x*) modes also characteristically redshift as *R* increases, it becomes hard to disentangle the individual effects.

To estimate the gap thickness for the NWoMs here, we experimentally obtain *R* from SEM images and mode positions from DF scattering and append those points to Figure 2e. The (1*x*) and (2*x*) mode positions of five such NWoMs vs AgNW radius are shown as orange and blue points, respectively. For one such NW, the (1*x*) position goes beyond 1000 nm which exceeds the capabilities of our spectrometer and therefore is not recorded in our measurements. The results are very close to the simulated resonance positions for *d* = 5 ± 0.6 nm, as expected from the PVP and BPT thicknesses.

We note that direct comparison of these 2D simulations with experiment is valid here, even though the illumination conditions are not exactly the same. For full 3D FDTD simulations, the same (1*x*) and (2*x*) modes appear with no additional modes seen in scattering. 2D simulations are however preferred due to rapid convergence for high-angle illumination, which is challenging given the computational requirements of such narrow gaps and large simulation volumes (Supporting Information, S2).

**Figure 3:**
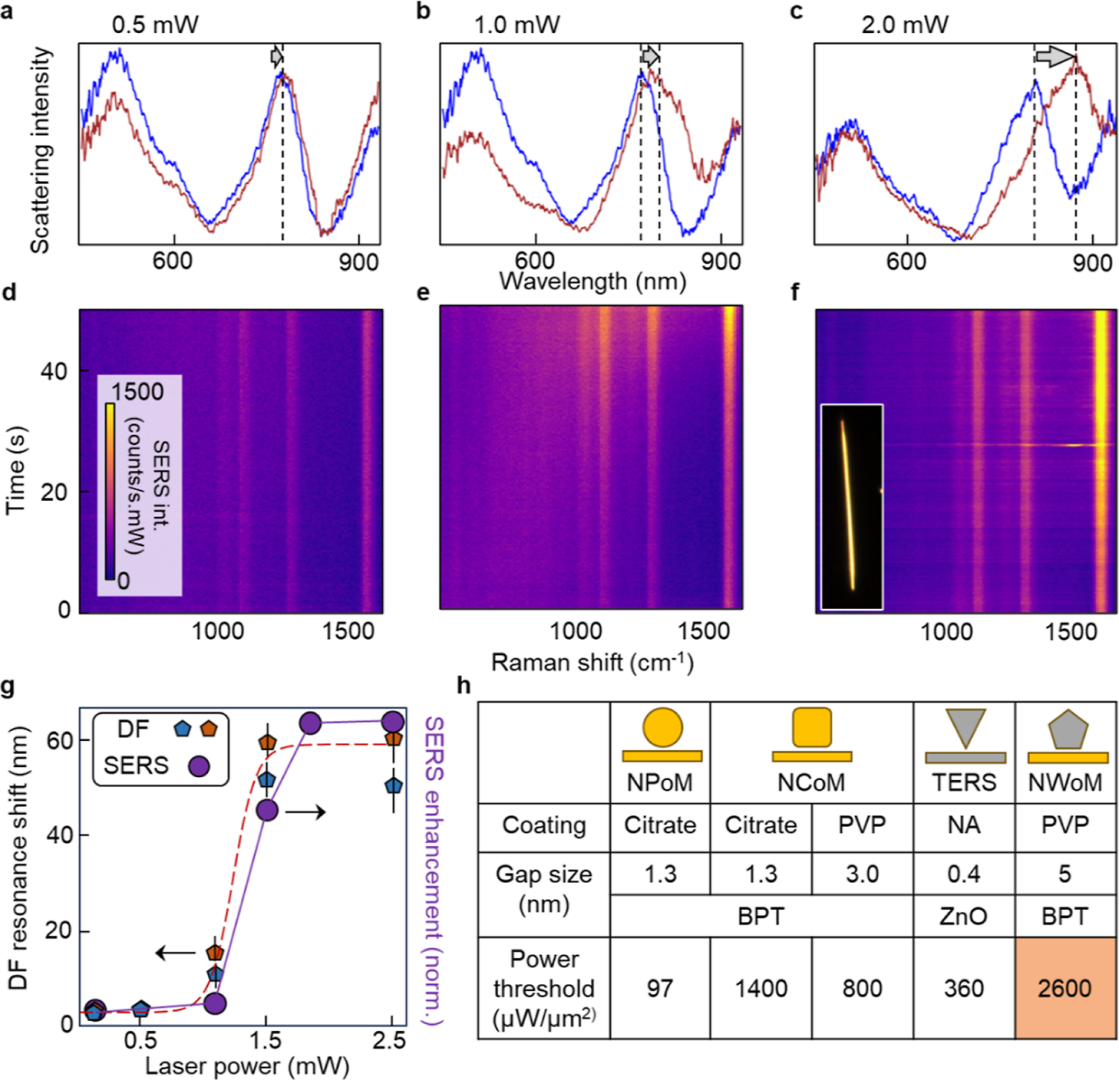
DF scattering spectra at different locations along the elongated length of the NWoM nanocavity for (a) *P* = 0.5 mW, (b) *P* = 1.0 mW, and (c) *P* = 2.0 mW. Black dotted lines show resonance position of NWoM nanocavity mode before and after the SERS time trace. Black arrow shows redshift of NWoM mode from *t* = 0 s (blue curve) to *t* = 50 s (red curve). SERS time traces for NWoM excited with (d) *P* = 0.5 mW, (e) *P* = 1.0 mW, and (f) *P* = 2.0 mW. Inset: optical image of NWoM used for measurements. (g) DF resonance shift and SERS enhancement vs laser power. Orange and blue points show reproducibility of DF shift for two different NWs. Lines show average DF shift (red dotted) and SERS intensity (purple solid) during time trace. (h) Table comparing power thresholds for surface atom diffusion into the gap for different plasmonic cavities with molecules sandwiched between the Au/Ag nanostructures and the Au mirror.

The near-field variations in the nanocavity alter both scattering and SERS intensities. For the specific NWoM in Figure 3, we estimate *d* = 5 nm and *R* = 40 nm. For this value of the NW radius, NWoM nanocavities exhibit a (1*x*) plasmon mode at around 800 nm and support a secondary (2*x*) plasmon mode closer to the chosen excitation laser wavelength. This (2*x*) plasmon mode ensures significant optical field enhancement within the nanogap, both at the excitation wavelength and at λ = 710 nm, corresponding to the SERS vibration lines for BPT molecules, leading to a strong SERS signal from the nanocavity (Supporting Information, S3). From the estimated gap size, we correlate SERS intensities with DF resonance shifts for each individual NWoM.

Four different locations along the extended length of a NW are probed with four different laser powers (*P* = 0.5–2.0 mW) for *t* = 50 s. DF scattering spectra are recorded at each location before and after laser exposure to track changes inside the nanogap. At each location, SERS time traces are recorded by taking 500 SERS spectra each with acquisition time of 100 ms. The calculated resonance position of the (1*x*) mode matches well with the experimentally obtained resonance position for *d* = 5 nm at *P* = 0.5 mW.

For this relatively low power of 0.5 mW, SERS time traces do not change over time (Figure 3d). The intense mode below 600 nm in Figure 3a represents the plasmonic mode of the AgNW and is evident in all the NWoM nanocavities (Supporting Information, S4). Since this mode does not correspond to a cavity mode, its spectral position is not sensitive to changes inside the cavity. While we could identify a (2*x*) mode at 600 nm, the signal is very weak and close to the intense plasmon peak of the AgNW.

The (1*x*) gap plasmon mode resonance position marked by the dotted black line does not shift from *t* = 0 s (blue curve) after *t* = 50 s (red curve) (Figure 3a).

This implies that at this power the gap thickness remains unchanged after laser exposure, and indeed the SERS intensity also does not change.

For *P* > 1.0 mW, the (1*x*) plasmon mode redshifts with time, and correspondingly the SERS intensity also increases (Figure 3b–f). A similar trend with the (1*x*) mode resonance shifting as a function of incident laser power at different locations along the nanocavity is seen for different NWs. The cavity mode redshift saturates at ∼60 nm above 2 mW (Figure 3g). Sigmoidal fits to the data show the same distinct power threshold (2600 μW/μm^2^) for the increase in SERS intensity.

The redshift in the (1*x*) mode and increase in BPT SERS both indicate a reduction in gap size, as opposed to a growth in the AgNW facet width. If the data were to be explained solely by a change in facet width, we would need an 8 nm increase in facet width to give a 55 nm shift in the DF, but this would result in an 85% decrease in the near-field (Supporting Information, S5). An increase in facet size along with an increase in the number of molecules probed thus does not explain the observed data. Only a local reduction in gap size can consistently explain the observed increase in BPT SERS signal.

We use this large redshift of the (1*x*) mode at high laser powers to estimate the change in gap thickness using simulations (Supporting Information, S6). The experimentally obtained resonance for *P* = 2.0 mW after *t* = 50 s matches the calculated resonance position corresponding to *d* = 2 nm. This suggests we observe atomic-scale morphological changes at room temperature inside the nanocavity that decrease the gap by ∼3 nm, purely from laser irradiation.

There can be several different possible reasons for this reduction in gap thickness inside the nanocavity. First is the melting of the PVP coating near the hot-spot inside the nanogap due to prolonged laser exposure. To estimate the temperature for the NW during laser exposure, we utilize Raman thermometry of plasmonic nanostructures. In our experiment, we calculate temperatures for each anti-Stokes time trace at a laser power *P* = 2.5 mW for the NWoM nanocavity (Supporting Information S7). Calculated temperatures are not significantly higher than room temperature, consistent with previous measurements, as the mirror underneath the NW acts as a heat sink and avoids thermal buildup.^29^ However, it is important to note that PVP undergoes a phase transition from a viscous glass to a rubber at a specific glass transition temperature (*T*_g_).^46^ For PVP, it has been shown that *T*_g_ depends on the molecular weight (MW).^47^ Here the NWs synthesized using a polyol process have PVP coatings with MW ranging between 25–55 kDa,^26^ corresponding to *T*_g_ around 380–400 K. Thus, our estimated temperatures for the NWoM during laser irradiation can exceed *T*_g_. This can lead to reorganization of the PVP polymer and infiltration with Ag atoms during laser exposure.

We propose that along with the reorganization of PVP, there is diffusion of surface Ag atoms near the hot-spot inside the nanocavity during high power laser exposure. This diffusion can be enhanced by the interaction of Ag atoms with PVP molecules heated >*T*_g_ due to their increased molecular mobility. As atoms diffuse into the gap, the gap thickness decreases and PVP atoms experience high optical near-fields, giving rise to additional PVP-Ag SERS lines (Supporting Information S8). It is evident from the SERS spectra (blue curve in Figure 1c) that PVP-Ag SERS lines appear when the diffusion starts. The new vibration modes (1300–1400 cm^–1^) correspond to a symmetric breathing mode of the PVP pyrrole ring and asymmetric deformation of the ring.^28,48^ Both modes involve C=O bond stretching with the oxygen atom attached to the Ag atom. We do not observe any peaks around 1650–1750 cm^–1^, which is a signature of free PVP molecules.^49^ Even at high powers, PVP remains attached to the Ag atoms but has diffused into the gap, experiencing enhanced near-fields and thus providing strong SERS signals. At high laser powers (>2 mW), SERS intensity counts saturate (>6 × 10^4^ counts) after long kinetic scans, suggesting a limit to the gap reduction (Supporting Information S9). This type of surface atom diffusion at room temperature, although transient, has been reported in the past for Au atoms in NPoM nanocavities.^19^ This is the first report to observe such restructuring of the surface in an elongated nanocavity, which we exploit here to further demonstrate spatial lithography. The formation of atomic-scale constrictions by light-driven diffusion of multiple atoms is supported by the critical power threshold, which shows when optical forces can exceed the energy barrier for surface atoms to diffuse into the gap. The resulting compressed nanogap accounts well for the increased near-field.^21^

A general concern with this type of atomic diffusion is its stability. Here, we compare the power threshold for adatom diffusion in other geometries (reported in the literature), such as the NPoM and nanocube-on-mirror (NCoM), to the NWoM geometry as well as techniques such as tip-enhanced Raman scattering (TERS)^50^ (Figure 3h). It is known that the NCoM exploits higher-energy^41^ crystal facets face-down on the mirror as compared to low-energy {111} facets for NPoMs, thus giving higher power thresholds.^23,51^ It was also reported that for the same NPoM geometry, the power threshold depends on the molecular SAM within the nanogap.^21^ Importantly, we find that NWoMs can be excited with much higher powers (2.5 mW/μm^2^) before atoms diffuse and SERS signals destabilize, in comparison to both NPoM (0.1 mW/μ m^2^) and NCoM (1.4 mW/μm^2^). We also note that NWs have a larger facet size compared to NPs. This can potentially enable the probing of molecules with extremely low Raman cross sections at high powers for long measurement times to obtain robust SERS signals and for nonlinear measurements.

A possible reason for this increased threshold could be the strong PVP binding to Ag atoms,^28,52^ which thus needs higher powers for Ag atom diffusion into the 5 nm gaps. Larger nanogaps here also result in lower field confinement and thus smaller optical forces. The role of PVP is important here in improving the stability. Comparing the PVP binding to Ag/Au atoms, the effect of ligand coatings around plasmonic metallic nanostructures on the optical forces required to drive atoms into the cavity needs further investigation. The elongated gap supported by this NWoM provides an additional channel for thermal-sinking along with the mirror, so the heat generated is not just localized at the laser illumination point.

**Figure 4:**
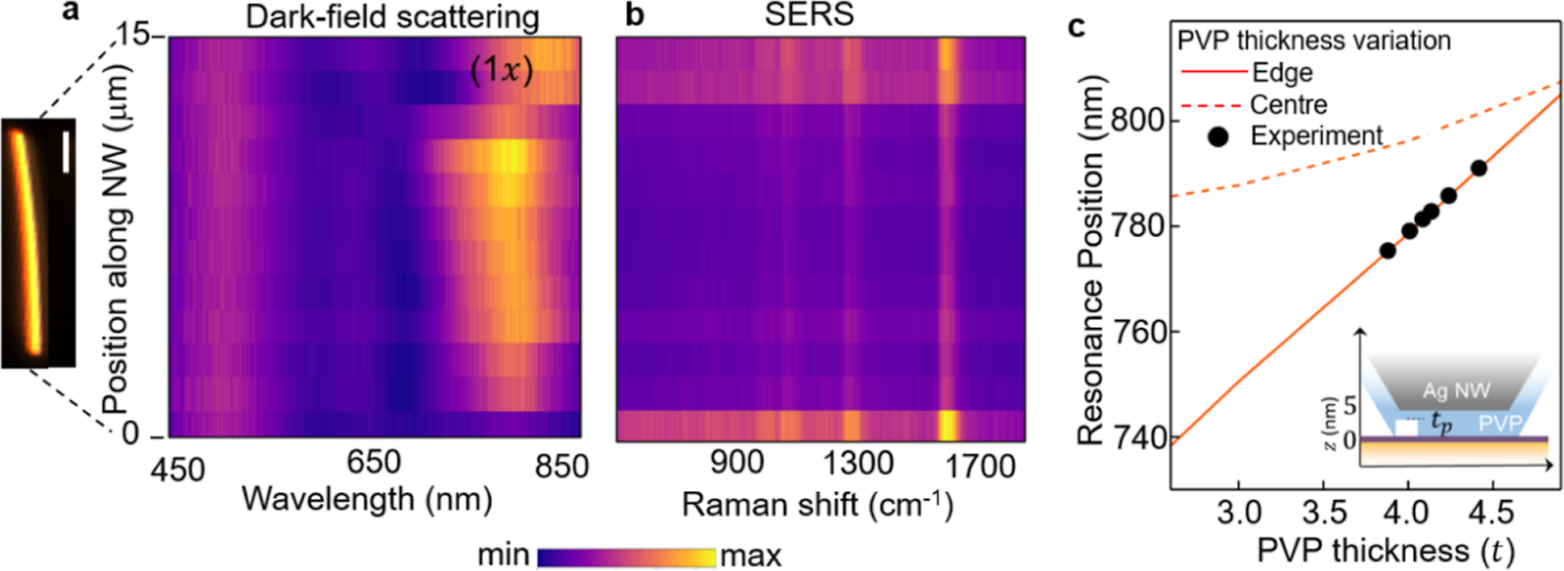
(a) Dark-field scattering spectra at different spatial positions along the nanowire. Inset: dark-field scattering image of the NWoM, scale bar = 100 nm. (b) SERS spectra along the same silver nanowire. (c) Simulated (1*x*) resonance position as a function of PVP thickness (*t*_p_) for NWoMs with fixed *d* = 5 nm nanogap (schematic shown in the inset). Orange solid and dotted lines represent thickness variation at the edge and center of the lower facet width, respectively. Black points are experimental data for resonance peak position along the length of the NW.

It is important to confirm that the spectral redshift observed for high-power continuous laser excitation exceeds any spectral shift caused by nonuniformities of the chemically prepared NWs.^27^ To confirm this, we record the variation of mode resonance along the length of the cavity without any laser excitation. This characterizes the variation of cavity gap size without any optical excitation. DF scattering is collected at 12 different points along the length of the NWoM (Figure 4a), with spatial resolution set by the ∼0.5 μm diffraction-limited spot of the excitation and collection fiber.

In DF scattering maps of the (1*x*) mode, redshifts are observed at both ends of the AgNW. It has been reported that synthesized AgNWs, along with their regular pentagonal geometry, possess needle-like cross sections at their ends (Supporting Information S10), depending on the molar ratio of PVP to silver nitrate used during chemical synthesis. These nonpentagonal cross-section edges and nonuniform PVP layer thicknesses change both the mode positions and in/out coupling of the modes, which also modifies the near fields. This variation is thus also reflected in the SERS intensities.

Along the length of the NW, the mode resonance position fluctuates. This must arise from nonuniformity of the gap contents with the mean peak centered at 778 nm, leading to corresponding fluctuations in SERS (Figure 4b). We observe a spectral wandering of the (1*x*) mode by ±10 nm from the average resonance wavelength of 778 nm. As indicated by the simulations, this small-scale variation is explained by <0.5 nm change in the gap thickness and can be attributed to sub-nm surface nonuniformities or nonuniform coating of PVP. This variation is simulated for a NWoM nanocavity with *R* = 45 nm and a fixed nanogap *d* = 5 nm (Methods). An air pocket (white rectangular box) with variable thickness *t*_p_ and refractive index *n*_t_ = 1 mimics the (sub-nm) PVP thickness variation inside the nanogap (inset Figure 4c). Two sets of simulations, with variability along the facet length and at the edges are shown (Figure 4c).

Because the optical field for the (1*x*) mode is concentrated at the NW edges (Figure 1d), the effect of *t*_p_ on the resonance position is minimal when under the NW center (dotted orange line) compared to the edge (solid orange line). Comparing the experimentally observed DF resonance positions (black points) suggests that the PVP coating thickness varies mostly near the NW edges. This also accounts for the weak but correlated dependence of SERS intensity on the (1*x*) spectral position. We suggest that these nonuniformities are due to sub-nm scale crystal facet changes at the NW edge.

**Figure 5:**
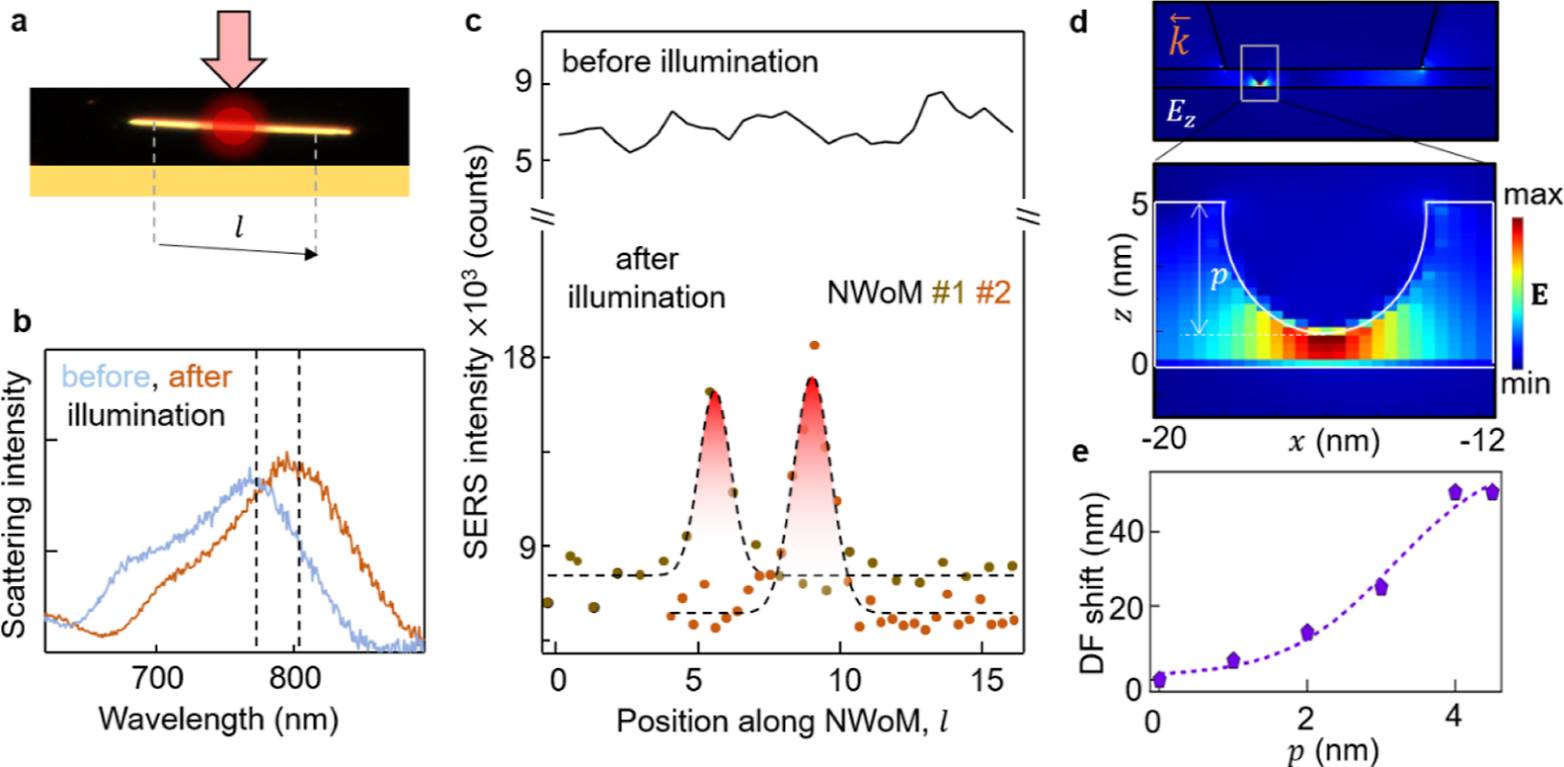
(a) Excitation halfway along the NW length (*l*) with a diffraction-limited laser spot at *P* = 2.5 mW. (b) DF scattering spectra from the excitation spot before and after illumination showing resonance mode redshift (dotted lines). (c) SERS intensities from a low-power laser (*P* = 1 mW) spatially scanned along the NW length before (black solid) and after central illumination (dotted curves). Highest signal at midposition. (d) Simulated optical near-field perpendicular to mirror (*E*_*z*_) for NWoM with an Ag sphere positioned midway along the NW facet. Zoomed-in image of white rectangular box showing Ag ellipsoid with radius *p* = 4 nm from Ag diffusion into the gap. (e) Variation of DF resonance shift as a function of *p*.

A specific location along the elongated gap is now exposed to high laser power (“write” *P* = 2.5 mW) compressing the gap size (Figure 5a). SERS emission from the NWoM cavity is collected along its length at low powers (below the threshold) both before and after the “write” laser excitation (Figure 5b). Before illumination, SERS intensities (*P* = 1.0 mW) are similar at different positions along the length (Figure 5c). After local modification from the “write” beam, SERS spectra are again collected from different positions along the NWoM nanocavity. In both NWs shown, the SERS spectra from the write position give double the SERS intensity compared to other positions. It is still unclear which wavelength would be most effective for localized writing, as both optical and thermal effects must be carefully considered along with the direction and polarization of the illumination. This necessitates experiments with tunable lasers, as resolution of this “writing” method will also depend on the wavelength. In this case under the same laser wavelength, for both NWs the changes inside the nanogap are local, nonreversible, and of similar magnitude.

We further verify using simulations that these morphological changes inside the nanocavity are local (Methods). To model the atom reconfiguration, we use a Ag sphere of radius *p* set by the diffusion length into the nanogap, positioned at a specfic location (*x* = −15 nm) along the facet of the NW for the NWoM nanocavity (Figure 5d). The optical profile shows enhanced near-field between the sphere and mirror (white rectangular box). The zoomed-in image clearly shows the field enhancement at the location where the atoms diffuse into the cavity, for *p* = 4 nm. The cavity compression results in an enhanced near-field which is very local to the excitation point. To correlate this diffusion with the shift in DF, we vary *p* and plot the corresponding shift in DF resonance mode (Figure 5e). It is convincing that for *p* = 4 nm, a DF resonance shift of around 60 nm is seen, which matches well with our experimental observations (Figure 3g). The local enhanced electromagnetic field around this protrusion also explains the enhanced PVP signatures observed in Figure 1b. To estimate the location of diffusion inside the facet-width, we simulate scattering cross-section variations with diffusion of atoms at different positions along the NW facet. As shown above, the (1*x*) resonance mode has enhanced near-fields at the facet edges, and restructuring at this edge results in (1*x*) resonance mode redshifts. Atom diffusion to the center does not affect the (1*x*) resonance mode so the spectra blueshifts (Supporting Information S11).

It is also important to confirm that BPT molecules remain attached to the Au mirror with no changes to the Au mirror. To confirm this, the NWoM nanocavity is excited with two laser wavelengths (λ = 633, 785 nm) simultaneously at the same location (Supporting Information S12). A low-power λ = 785 nm laser excites the NWoM system to act as a probe beam to scan the changes made by a high laser power (*P* = 2.5 mW) at λ = 633 nm. Increased SERS intensity from λ = 633 nm can be seen, along with consistent BPT SERS lines for λ = 785 nm even at *t* = 50 s. This confirms the change in near-field from restructuring of a nanogap without damaging the BPT molecules, thus providing consistent SERS from the nanocavity.

## Conclusion

In conclusion we show the local restructuring of a NW surface through the formation of stable atomic scale construction at room temperature in an elongated nanocavity with a high threshold power. This restructuring is ideal for obtaining strong robust SERS at high powers from molecules sandwiched inside the nanocavity and can also be simultaneously exploited to demonstrate spatial lithography under ambient conditions. Using DF scattering and SERS scattering from molecules, we map the local enhanced fields inside the nanogap. The PVP coating and its binding to the Ag atoms on the surface plays a significant role in determining the power threshold. These results indicate the possibility of controlled structural changes on the order of ∼3 nm inside an extended nanocavity of NWoM geometry. Such on-the-fly control of plasmonic tuning should allow the creation of plasmon cavity waveguides, networks, and coupled resonators.

## Methods

### Sample Preparation

Chemically prepared AgNWs are centrifuged first in acetone and then in ethanol to remove excess PVP and Ag NPs in the solution. The AgNWs are then dispersed in ethanol. For SAM formation, template-stripped Au substrates are immersed in a 1 μL solution of BPT in ethanol for 24 h. After this period, the substrate is removed from the BPT solution, washed with ethanol to remove excess molecules, and a 5 μL solution of AgNW is drop-cast on the SAM-coated Au substrate and dried with nitrogen. We estimate the total gap thickness (PVP + BPT) for NWoM to be 5 nm from our DF measurements. From the previous extended set of experiments, the size of the BPT monolayer on mirror has been calculated to be around 1 nm. Thus, we estimate the size of PVP thickness to be around 4 nm for our NWoM nanocavities.

### Experimental Setup

Samples containing AgNWs on an Au mirror are placed on a computer-controlled motorized stage on an Olympus BX51 microscope. A 100 × 0.9 NA objective lens is used with a halogen lamp and scattered light is collected using the same lens. The scattered light is split between a fiber-coupled (Ocean Optics QEPRO) spectrometer and a Lumenera Infinity3-1 camera for DF spectroscopy and imaging. For SERS, a particular location on the NW is excited with a diffraction-limited spot from a 633 nm wavelength diode laser (Matchbox) after passing through a laser line filter. A half-wave plate in the excitation path controls the polarization state of the incident light. Collected backscattered light is focused on the 300 lines/mm grating of an Andor Shamrock i303 spectrograph using a tube lens, and elastically scattered light is rejected using two notch filters. For polarization-resolved measurements, an analyzer is placed in both DF and SERS paths just before each spectrometer. For Figure 2b, where correlated SERS measurements are not required, scattered light from the sample was sent directly to a fiber-coupled spectrometer. With only an analyzer in the optical path, this setup resulted in better signal-to-noise in the DF spectra compared to Figure 3. For measuring SERS spectra throughout this work, the laser spot is focused at the center of the NW avoiding the NW edge. AgNWs edges have complex geometries giving rise to inconsistent results.

### Numerical Simulations

Figure 1: Lumerical FDTD Solutions v8.12 was used for 2-D simulations of near-electric field inside the nanogap for the NWoM system. The AgNW is modeled using a pentagonal geometry (*xz* plane) with refractive index set to Ag. A rectangular geometry with a thickness of 500 nm and refractive index of Au models the Au substrate. A nanogap (*d* = 5 nm) between the pentagonal geometry NW and Au mirror with refractive index of 1.4 models the PVP coating. The geometry was excited with a Gaussian beam polarized perpendicular to the NW (*z*-direction) with λ = 633 nm. The meshing size of the whole geometry is set to 3 nm along both *x*- and *z*-axes. The nanogap (along *z*-axis) is meshed with 0.5 and 1 nm meshing size for the nanogap along the perpendicular direction and along the NW length, respectively. A frequency domain field monitor was used to calculate enhanced electric field inside the gap in the *x*- and *z*-direction.

Figure 2: The NWoM system is modeled similarly to Figure 1 with a varying nanogap (*d* = 3, 5 nm) between the NW and Au mirror. For calculating the scattering cross-section, the full geometry is excited using a total field scattered field source. The scattering cross-section monitor, along with a frequency domain monitor, was used to calculate the scattering cross-section and map the near field for DF resonances.

Figure 4: To model the nonuniformities of PVP surface coating, a rectangular box with variable thickness “*t*_p_” and refractive index = 1 was placed inside the nanogap for NWoM geometry. Two sets of scattering cross-section calculations were performed for the box at the center and at the edge. For both positions, the thickness of the box was varied and the resonance position for the (1*x*) mode was recorded.

Figure 5: To simulate picocavities, an ellipsoid with a refractive index matched to Ag was placed inside the nanogap (around the edge, *x* = −15 nm for NWoM nanocavity with *R* = 30 nm). Radius 1 (=2 nm) of the ellipsoid was kept constant. Radius 2 “*p*” which shows the diffusion of atom into the nanogap, was varied from 0 to 4.5 nm. The scattering cross-section monitor was utilized to calculate the redshift of the resonance mode with a frequency domain monitor to map the near-field (*E*_*z*_) inside the gap.

### Laser Power Density

To estimate the laser power density, we calculate the laser spot-size for a 633 nm laser focused using a 0.9 NA objective lens. The experimental value of the radius of the spot-size is calculated to be *r* = 490 nm with the laser spot area (*A* = π*r*^2^) to be around 0.753 μm^2^. For the laser power threshold *P* = 2 mw, the laser power density = P/A is estimated to be around 2600 μW/μm^2^.

## Supporting Information Available

(S1) Optical setup for dark-field and SERS measurement. (S2) Comparison between 3- and 2-D FDTD simulation for scattering cross-section. (S3) Simulated optical near-field (*E*_*z*_) profiles for NWoM with *R* = 45 nm (S4) DF scattering spectra of the NW with *R* = 30 nm placed on glass and gold substrate. (S5) DF resonance shift as a function of facet-width increase. (S6) Correlation between variation of (1*x*) mode position vs change in gap thickness. (S7) Temperature measurement for the NWoM nanocavity system during laser exposure. (S8) Peak assignment for PVP-metal SERS peaks. (S9) Saturating SERS intensity from NWoM nanocavity. (S10) SEM images of AgNWs with needle-like cross-section. (S11) DF variation with atomic restructuring at different positions along the NW facet. (S12) Dual laser excitation for NWoM nanocavity (PDF)
