# Supplementary material for: Mapping and Optically Writing Nanogap Inhomogeneities in 1-D Extended Plasmonic Nanowire-on-Mirror Cavities
Source: ACS Photonics. 2024 Dec 10;11(12):5205–14. doi: 10.1021/acsphotonics.4c01443 (PMC11660218; doi:10.1021/acsphotonics.4c01443)
Supplement: Supplementary file 1 — ph4c01443_si_001.pdf [file ph4c01443_si_001.pdf]

Supporting Information

Mapping and optically-writing nanogap inhomogeneities in 1-D extended plasmonic nanowire-on-mirror cavities

Chetna Taneja[1,2], Eoin Elliott[1], G V Pavan Kumar[2], Jeremy J Baumberg[1] and Rohit Chikkaraddy[3*]

[1] NanoPhotonics Centre, Cavendish Laboratory, Department of Physics,
JJ Thompson Avenue, University of Cambridge, Cambridge, CB3 0HE, United Kingdom

[2]Department of Physics,
Indian Institute of Science Education and Research Pune, 411008, India

[3]School of Physics and Astronomy, University of Birmingham, Birmingham B15 2TT, United Kingdom

S1: Optical set-up for dark-field (DF) and surface enhanced Raman scattering (SERS) measurement

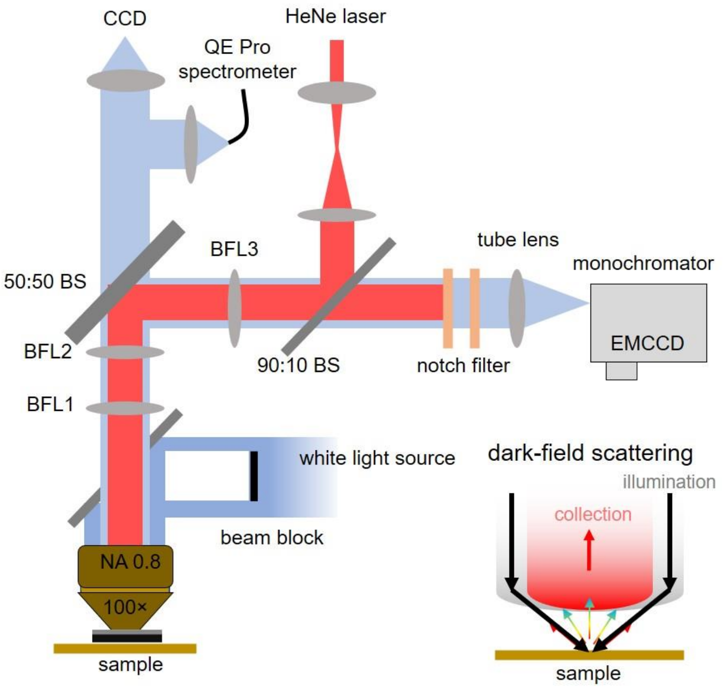

Fig. S1. Optical set-up used for the DF scattering spectra and SERS measurement using DF microscope equipped with a computer controlled linear stage. White light was focussed on the sample using DF objective lens 100x 0.9, and the back-scattered light was collected. Scattered light was sent into CCD for imaging and into a fibre coupled spectrometer using beam-splitters in the path. Lens was used to focus the scattered light into the spectrometer and a polariser was placed just after the lens for the polarisation resolved DF scattering spectra. All the spectra were background subtracted and normalised with respect to the scattering off a broadband Lambertian scattering plate to compensate for chromatic aberrations in the optics and spectrometer. For SERS measurements, 632.8 nm He-Ne laser was focused at a certain position on the sample using the same objective lens and the scattered light was sent into spectrometer after passing through two 633 nm edge filters to block the elastically scattered light. A half wave plate (HWP) was placed in the incoming path of the laser to control the

laser polarisation with respect to nanowire (NW) axis. To maintain the polarisation set by HWP, a plate beam splitter used in the incoming path was placed such that laser hits the beam-splitter at an angle close to critical angle.

S2: Comparison between 3-D and 2-D FDTD simulation for scattering cross-section

3-D simulation with nanowire-on-mirror (NWoM) nanocavity is computationally expensive. In the main manuscript all results obtained using 2-D simulations are presented. However, we simulate scattering cross-section for one AgNW with radius R= 30nm and 10μm length placed on Au mirror using 3-D FDTD simulations. As can be seen that relative position of both $(1x)$ and $(2x)$ modes are shifted by few nanometres in the case of 3-D simulations which can be attributed to the poor meshing of the nanogap due to computational size limitations. However, it is evident that there are no additional plasmon modes and 2-D simulations are sufficient to corroborate with experimental results.

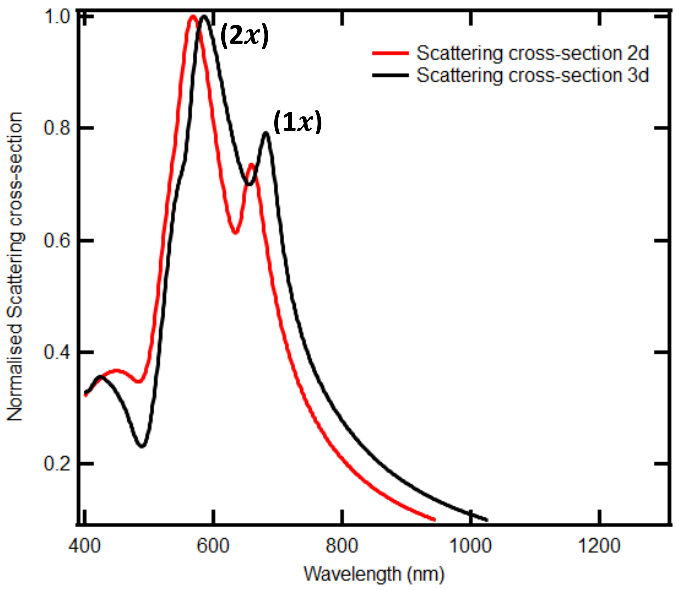

Fig. S2. Normalised scattering cross-section for AgNW obtained using 3-D (black curve) and 2-D FDTD simulation (red curve).

S3: Simulated optical near-field (E_z) profiles for NWoM with R= 45nm

Fig. S3 shows two simulated near-electric field profiles for NWoM with R= 45 nm at the 633 nm excitation wavelength and wavelength corresponding to the most intense SERS peak for BPT molecules (= 710 nm at 1585 cm^{-1}). Given the field is confined inside the nanogap at both excitation and SERS wavelengths, BPT provides robust SERS signals from the nanocavity at our chosen wavelength.

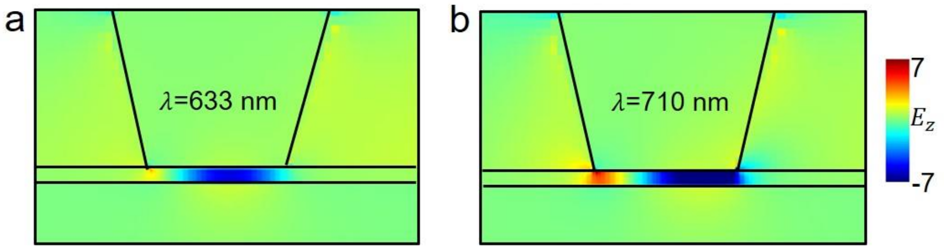

Fig. S3. (a) Simulated optical near-field (E_z) profiles for NWoM with R= 45nm at the excitation wavelength (λ= 633 nm) and at the wavelength corresponding to the most intense SERS peak of BPT molecules (at 1585 cm^{-1}).

S4. DF scattering spectra of the NW with R= 30nm placed on glass and gold substrate

The mode below 600 nm is the plasmon mode of Ag nanostructures and is evident for all NWoM nanocavities. Fig. S4 shows the simulated DF scattering cross-section for a Ag NW (radius= 30 nm) placed on a glass substrate (black curve) and the gold mirror (red curve). The mode below 600 nm represents plasmon mode for Ag NW over glass (NWoG) geometry whereas NWoM nanocavity supports cavity plasmon modes. Another important point to note is that while cavity modes ($2x$) and ($1x$) are very sensitive to nanogap thickness, the mode below 600 nm does not shift spectrally.

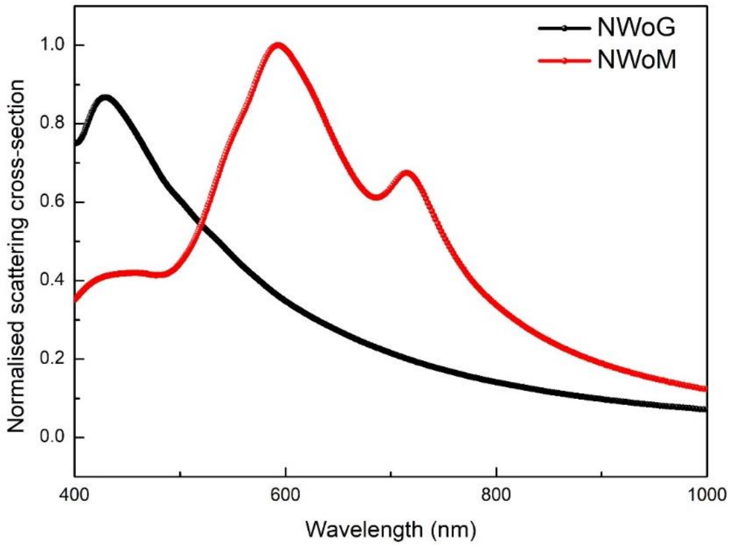

Fig. S4. DF scattering spectra of the AgNW with radius R= 30nm placed on glass (black curve) and gold mirror (red curve) forming NWoM nanocavity.

S5: DF resonance-shift as a function of facet-width increase

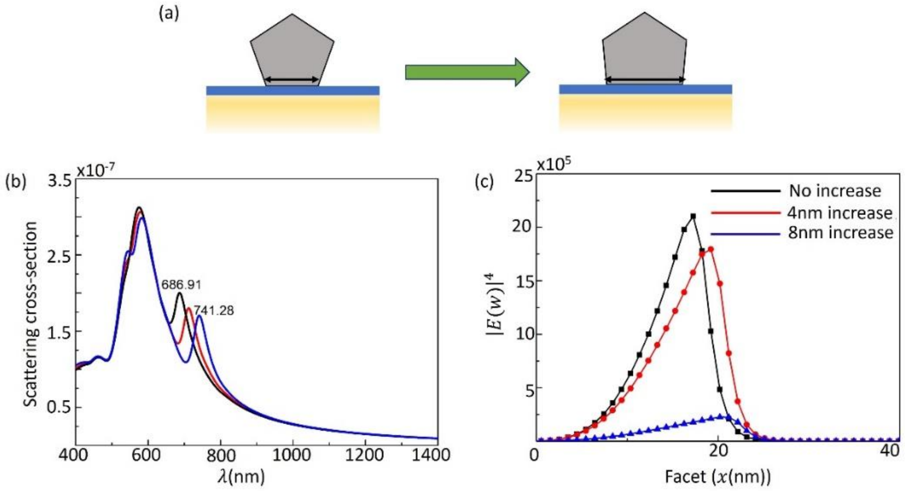

Fig. S5 (a) Schematic of NWoM nanocavities for AgNW (R= 30nm) with regular pentagonal cross-sssection to cross-section with increased facet-width maintaining constant nanogap d= 5nm. (b) Simulated scattering cross-section for regular pentagon (black curve), total facet width increased by 4nm (red curve) and 8nm (blue curve). For DF mode ($1x$) shift of the order of 55nm, facet width must increase by more than 8nm. (c) SERS enhancement (fourth power of near-electric field inside the nanogap (d= 2.5 nm)) at λ= 689nm for regular pentagon (black curve), total increment in facet width by 4nm (red curve) and 8nm (blue curve).

S6: Correlation between variation of $(1x)$ mode position vs change in gap thickness

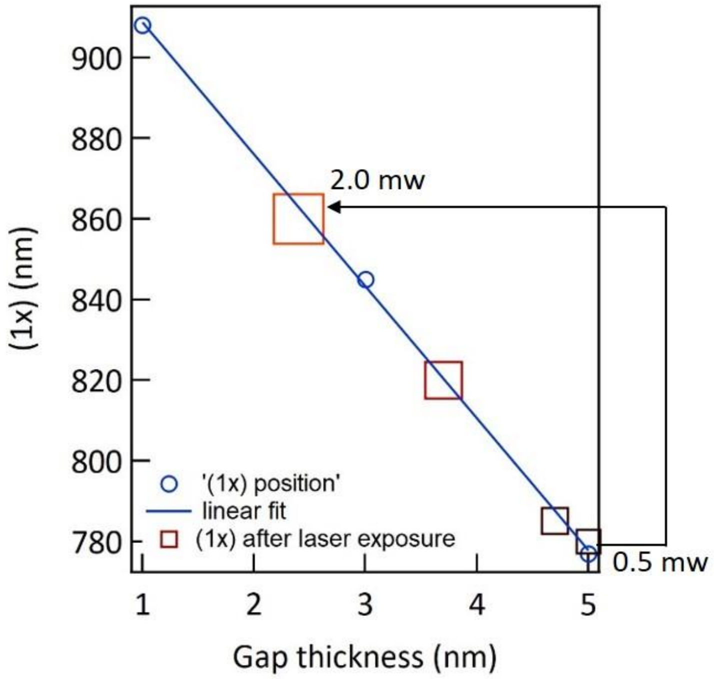

Fig.S6 $(1x)$ resonance mode position for NW with R= 40 nm placed on Au mirror with variable gap thickness (blue circles). Solid blue line represents the linear fit to the data. Squares with colormap 'hot' shows the resonance position after the laser excitation. Size of the squares are proportional to incident power with smallest square showing the $(1x)$ mode position after incident laser exposure with P= 0.5 mw. It is to note that experimentally obtained resonance position for P=2.0 mw after the SERS time trace is closer to the calculated resonance position corresponding to d= 2.5 nm. This data strongly suggests the local morphological modifications in the gap thickness (~ 2 nm) dependent on the power of incident light.

S7: Temperature measurement for the NWoM nanocavity system during laser exposure

For an estimation of the temperature, we utilise Raman thermometry of plasmonic nanostructures. It has been shown that anti-stokes SERS background intensity exhibits exponential decay given by the thermal distribution of electron-hole pairs in metals with occupancy n given by

$$n = \frac{1}{\exp\left(\frac{-\Delta E}{k_B T}\right) - 1}$$

Here, ΔE is the energy difference between the excitation laser and inelastically scattered light, k_B is the Boltzmann constant.

The strong dependence on temperature provides a non-invasive tool for measuring temperature of plasmonic nanostructures using anti-stokes SERS exponential decay at a particular temperature. This equation can be modified to get rid of wavelength dependent scattering due to plasmon resonances of NWs in wavelength range of interest. Normalisation of the anti-stokes intensity (I_{au}) at a particular temperature 'T_{au}' with intensity (I_0) at room temperature (T_0) can be given as:

$$\frac{I_{au}}{I_0} = \frac{\exp\left(-\frac{hc\Delta\omega}{k_B T_0}\right) - 1}{\exp\left(-\frac{hc\Delta\omega}{k_B T_{au}}\right) - 1}$$

By taking the ratio of intensities at anti-stokes wavelength at a given time with intensities at room temperature and fitting the above equation provides Tau for NWoM nanocavity. For our experiment, anti-stokes time trace for a laser excitation of P= 2.5 mw for NWoM nanocavity is given in Fig. 4(a) and the calculated temperatures for each SERS spectra in the time trace as a function of time is given in fig. 4(b).

It can be seen from the graph that average temperature value of the NWoM system does not exceed beyond 300K. The calculated values of temperatures are not high enough to melt the PVP coating on AgNW.

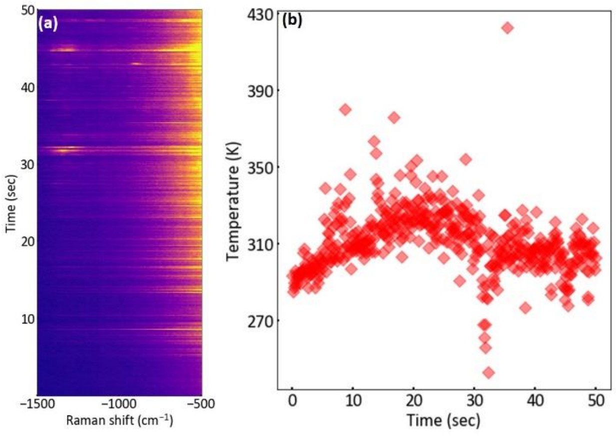

Fig. S7 (a) SERS anti-stokes time trace for laser power P= 2.5 mw at a fixed location along the NWoM nanocavity. (b) Calculated temperature (in kelvin) of the NWoM system from the anti-stokes SERS spectra as a function of time.

S8: Peak assignment for PVP-metal SERS peaks

Previously published report (J. Phys. Chem. C 2014, 118 (31), 17956-17967, ref [28] in main manuscript) provided assignment of main Raman SERS features from the DFT calculations for PVP attached to a metal atom. The reported peaks assignment clearly indicates that PVP-metal SERS peaks lie in the 1150-1450 cm $^{-1}$ region which matches with our observations. Below, we tabulate the peak assignments from this study.

Measured Raman Frequency (cm^{-1})	PVP Peak Assignment
1023	CH_2 rocking
1296	CH_2 wagging, C=N stretching
1342	symmetric pyrrole cycle breathing
1450	deformation of the pyrolle cycle (C-N-C)
1494	C=N stretching

Table T1: Tabulating the peaks assignment showing PVP-metal SERS peaks lie in the 1150-1450 cm $^{-1}$ region [data produced from ref 28 of main manuscript].

S9: Saturating SERS intensity from NWoM nanocavity

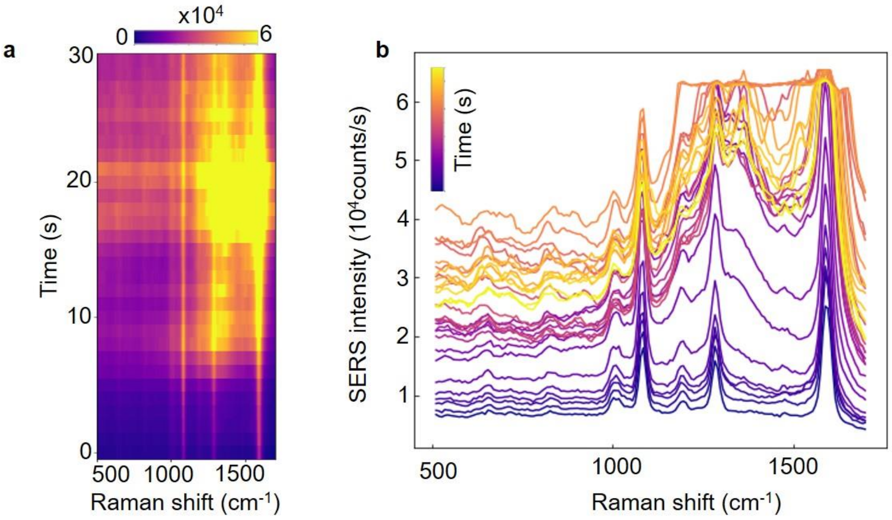

Fig. S8 (a) SERS time-trace for with laser excitation λ= 633 nm at a specific location along the length for NWoM nanocavity with laser Power P= 2.5 mw and acquisition time t=1s. Saturation in the SERS intensity counts can be seen around time t= 20s. (b) SERS spectra as a function of Raman shift (cm^{-1}) where the colorbar represents the spectra ranging from time t=0-30s.

S10: SEM images of AgNWs with needle-like cross-section

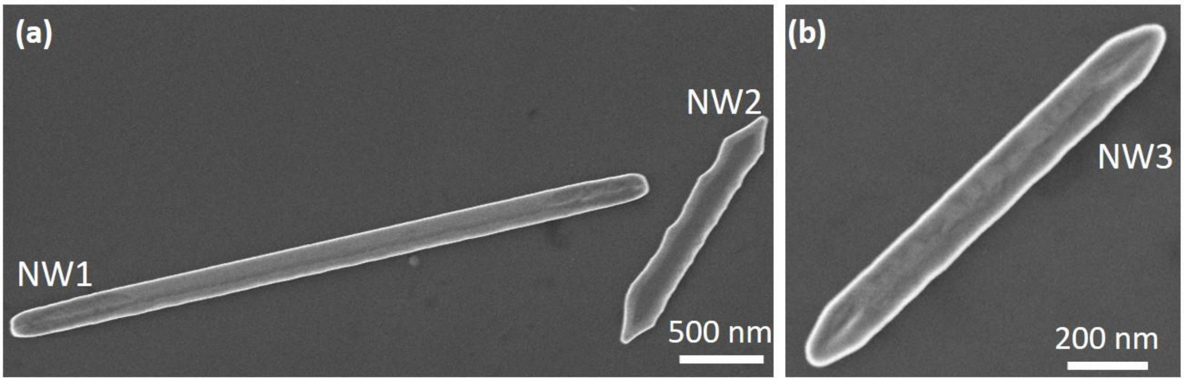

Fig. S9 (a)-(b) SEM images of AgNWs placed on a glass substrate with regular pentagon cross-section (NW1) and needle-like non pentagonal cross-section (NW2, NW3) from the same solution.

S11: DF variation with atomic restructuring at different positions along the NW facet

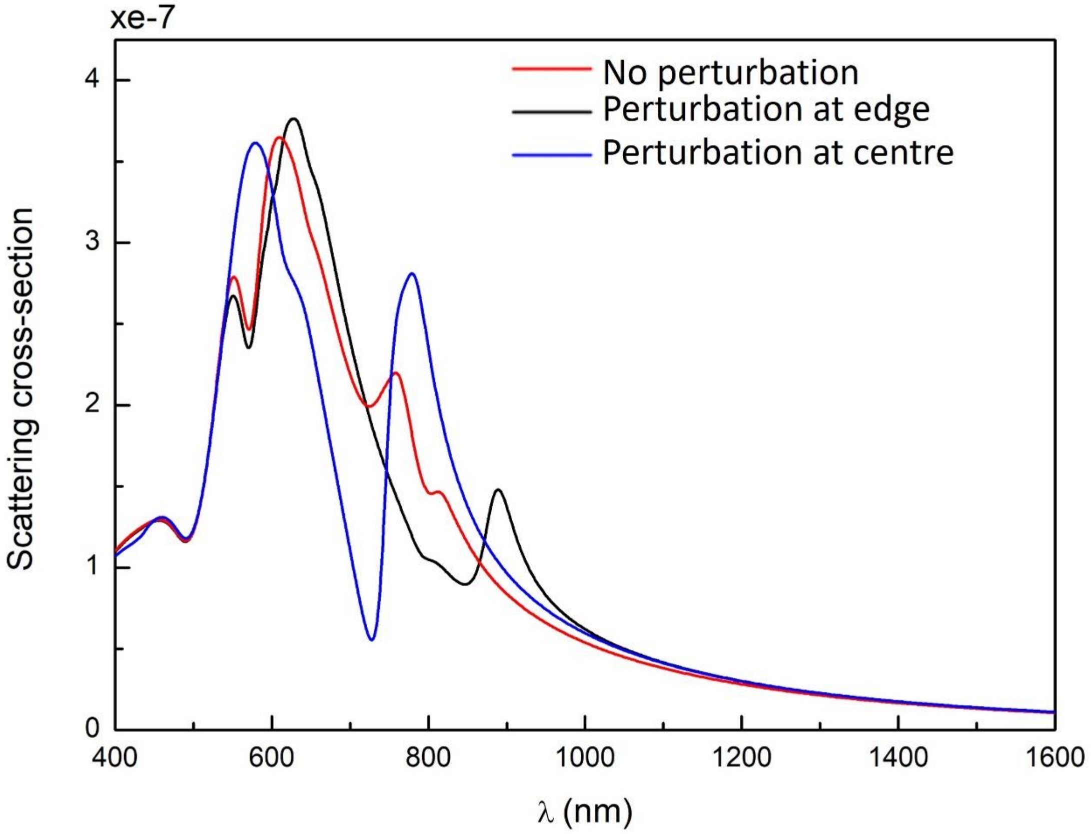

Fig. S10 Scattering cross-section with no atomic restructuring (perturbations with atom diffusing inside the nanogap) along facet-width (red curve), perturbations at the edge of facet-width (black curve) (c) at the centre of the facet-width (blue curve). Since $(1x)$ resonance mode has enhanced near-field at the edge of the facet, restructuring at the facet-end results in $(1x)$ resonance mode red-shift. Scattering cross-section for atom diffusing at the centre does-not show $(1x)$ resonance mode and spectra blue-shifts.

S12: Dual laser excitation for NWoM nanocavity

It is also important to confirm that structural changes which are decreasing the gap size and result in high SERS intensity from NWoM nanocavity are due to atoms diffusing from AgNW and BPT molecules are not damaged during the laser exposure. To confirm, NWoM nanocavity is excited with two laser wavelengths (λ= 632.8 nm, 785 nm) simultaneously at the same location (Fig. S7(a)). A low power λ= 785 nm laser excites the NWoM system to act as a probe beam to scan the changes made by a high laser power (P= 2.5 mw) of λ= 632.8 nm.

SERS time trace is collected for a total of t= 50 sec. BPT SERS from λ= 632.8 nm (red curve) and λ= 785 nm (purple curve) at time t=0 sec (Fig. S7(b)) shows high SERS intensity for λ= 632.8 nm. This can be attributed to low power and poor collection efficiency of the detector at the wavelengths corresponding SERS lines for λ= 785 nm excitation. Fig. S7(c) shows the BPT SERS from λ= 632.8 nm (red curve) and λ= 785 nm (purple curve) at time t=50 sec. Increased SERS intensity for SERS lines from λ= 632.8 nm excitation can be seen along with the consistent BPT SERS lines for λ= 785 nm at

t=50 sec. The molecules are still attached to Au mirror and thus providing consistent SERS from the nanocavity.

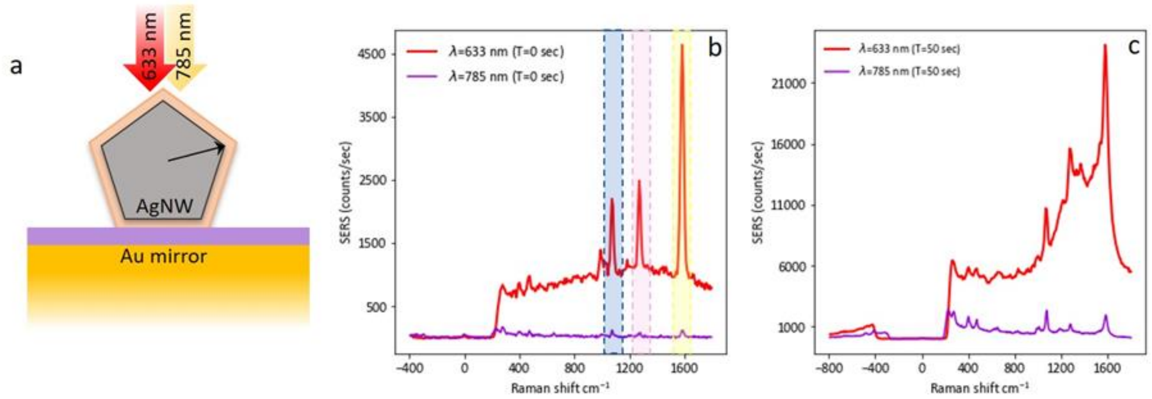

Fig. S11 (a) Schematic of NWoM nanocavity with dual wavelength excitation. Two lasers with high power (P= 2.5 mw) 632.8 nm laser wavelength and low power 785 nm laser wavelength are exciting the NWoM system using the same objective lens in upright microscope configuration. (b) SERS spectra collected at time (t= 0 sec) with excitation laser λ= 632.8 nm (red curve) and λ= 785 nm (purple curve). (c) SERS spectra collected at time (t= 50 sec) with excitation laser λ= 632.8 nm (red curve) and λ= 785 nm (purple curve).
